# Decarburization of Wire-Arc Additively Manufactured ER70S-6 Steel

**DOI:** 10.3390/ma16103635

**Published:** 2023-05-10

**Authors:** Aprilia Aprilia, Wengang Zhai, Yibo Guo, Aishwarya, Robert Shandro, Wei Zhou

**Affiliations:** 1 School of Mechanical and Aerospace Engineering, Nanyang Technological University, 50 Nanyang Avenue, Singapore 639798, Singapore; aprilia@ntu.edu.sg; 2 Singapore Centre for 3D Printing, School of Mechanical and Aerospace Engineering, Nanyang Technological University, 50 Nanyang Avenue, Singapore 639798, Singapore; wengang001@e.ntu.edu.sg (W.Z.); yibo002@e.ntu.edu.sg (Y.G.); 3 Cetim-Matcor Technology & Services Pte. Ltd., 3 Seletar Aerospace Link, Singapore 797550, Singapore; aishwarya@cetim-matcor.com (A.); robert.shandro@cetim-matcor.com (R.S.)

**Keywords:** decarburization, wire arc additive manufacturing, ER70S-6, low carbon steel, heat treatment, simulation

## Abstract

Decarburization is an unwanted carbon-loss phenomenon on the surfaces of a material when they are exposed to oxidizing environments at elevated temperatures. Decarburization of steels after heat treatment has been widely studied and reported. However, up to now, there has not been any systematic study on the decarburization of additively manufactured parts. Wire-arc additive manufacturing (WAAM) is an efficient additive manufacturing process for producing large engineering parts. As the parts produced by WAAM are usually large in size, the use of a vacuum environment to prevent decarburization is not always feasible. Therefore, there is a need to study the decarburization of WAAM-produced parts, especially after the heat treatment processes. This study investigated the decarburization of a WAAM-produced ER70S-6 steel using both the as-printed material and samples heat-treated at different temperatures (800 °C, 850 °C, 900 °C, and 950 °C) for different durations (30 min, 60 min, and 90 min). Furthermore, numerical simulation was carried out using Thermo-Calc computational software to predict the carbon concentration profiles of the steel during the heat treatment processes. Decarburization was found to occur not only in the heat-treated samples but also on the surfaces of the as-printed parts (despite the use of Ar for shielding). The decarburization depth was found to increase with an increase in heat treatment temperature or duration. The part heat-treated at the lowest temperature of 800 °C for merely 30 min was observed to have a large decarburization depth of about 200 μm. For the same heating duration of 30 min, an increase in temperature of 150 °C to 950 °C increased the decarburization depth drastically by 150% to 500 μm. This study serves well to demonstrate the need for further study to control or minimize decarburization for the purpose of ensuring the quality and reliability of additively manufactured engineering components.

## 1. Introduction

Decarburization is a carbon-loss process in a material that occurs due to its exposure to oxidizing environments at elevated temperatures [1]. At high temperatures, the diffusion rate of carbon is so large that a significant amount of carbon loss may occur in regions adjacent to the surface [2,3]. Decarburization results in poor material properties, such as lower strength, poor wear resistance, and reduced fatigue life [4,5,6]. Decarburization remains a persistent problem in the manufacturing industry for processes that involve high-temperature heat treatment of steels [7].

In our previous studies, we investigated the effect of laser surface hardening processes on the decarburization of three different steels [8,9]. It was found that although the laser surface hardening process is fast, decarburization still occurs on the surfaces of AISI 1055 plain carbon steel and 420 martensitic stainless steel. However, for the 50CrMo4 alloy steel, decarburization was found to be negligible due to the presence of large amounts of alloying elements that may hinder the carbon diffusion process [8]. This shows that decarburization is a complex phenomenon that may or may not occur during laser surface hardening processes, depending on the specific type of steel.

Some studies have reported the decarburization of parts produced by additive manufacturing (AM) processes such as laser powder-directed energy deposition (L-DED) [10] and laser powder bed fusion (L-PBF) [11,12,13]. Lee et al. [10] reported decarburization in a heat-treated FeCrV L-DED sample and attributed the tensile surface residual stress to it. Mathias et al. [11] observed that the L-PBF sample heat-treated at 1085 °C in the air had a gradation of structures from the edges to the center due to decarburization. Zhao et al. [12] observed decarburization in 420 stainless steel L-PBF samples and attributed the lower Young’s modulus and hardness at the molten pool boundary to the decarburization. Seede et al. [13] observed decarburization of the AF9628 L-PBF sample and attributed the lower ultimate tensile strength and Charpy impact toughness to it. However, it should be noted that the decarburization reported by Zhao et al. [12] and by Seede et al. [13] is bulk decarburization throughout the printed microstructures (in contrast to the usual decarburization which occurs in the surface layers) when metallic powders were deposited during the L-PBF processes, and they concluded the decarburization mainly by comparing the carbon contents of powder feedstocks and as-printed samples.

Currently, there is a lack of systematic study on the decarburization of AM-produced parts, especially on the decarburization layer observation and depth evaluation, as well as the influence of different heat treatment temperatures and soaking durations on the resulting decarburization depths. Therefore, this study aimed to investigate these three areas systematically. So far, there has been no study on decarburization during wire arc additive manufacturing (WAAM) processes, so WAAM-produced parts were used in the current study.

WAAM is a material build-up process whereby metal wires are used as the material feedstock and an electric arc energy source is used for the fusion process [14]. It is a configuration of directed energy deposition (DED) technology that is both cost-effective and time-efficient [15]. It is an extended application of arc welding technology in building components using a layer-by-layer strategy [16]. WAAM is uniquely suitable for producing large engineering parts and has been widely used in the aerospace, maritime, and oil and gas industries [17,18].

It is important to study the decarburization of WAAM-printed parts because such parts are often exposed to high temperatures during the production and post-production heat treatment processes. Furthermore, as the WAAM-produced parts are usually large in size, the use of a vacuum environment to prevent decarburization is not always feasible [19]. Therefore, it will be useful to study the decarburization of WAAM-produced parts, especially after the heat treatment processes. Post-production heat treatment is an essential step in the WAAM process as it can be used to optimize or homogenize the microstructure and mechanical properties of the material [20,21].

In this study, the decarburization of a low-carbon steel ER70S-6 produced by a WAAM process was investigated. Both as-printed and heat-treated samples were evaluated. Heat treatments at various temperatures (800 °C, 850 °C, 900 °C, and 950 °C) and soaking durations (30 min, 60 min, and 90 min) were carried out. Decarburization characterization is ideally conducted by carbon measurement. However, carbon measurement is highly challenging. The usual chemical composition measurement technique, energy-dispersive X-ray spectroscopy (EDS), is not able to measure the carbon content accurately. Due to the high background count in an EDS machine, there is always an artificial carbon peak detected. Furthermore, EDS cannot measure low atomic number elements such as B, C, N, and O accurately. The accuracy of the carbon measurement is too low for decarburization evaluation. Even with advanced equipment, measuring carbon change is still challenging, especially for low-carbon steel. This has been hindering the decarburization research’s progress. Therefore, in this study, to evaluate the decarburization of WAAM-produced parts, microstructure and microhardness characterization methods were utilized instead. The approach is based on the guidelines from the ASTM E1077-14 standard [22]. In addition, to obtain the most accurate estimation of the decarburization depths, a water-quenching method was utilized after the heat-treatment processes to make the decarburization layer more distinguishable in the hardness profile. Numerical simulations of the carbon concentration profiles of the low-carbon steels during the heat-treatment processes were also carried out using the computational software Thermo-Calc 2023a (Thermo-Calc Software, Stockholm, Sweden).

## 2. Experimental Procedures

### 2.1. WAAM Process

The WAAM process was carried out using a RoboWAAM machine built by WAAM3D (Milton Keynes, UK). It is an integrated WAAM machine that uses a KUKA 6-axis anthropomorphic robot and a KUKA 2-axis servo positioner for the motion system. EWM Tetrix 552 AC Synergic was used as the power source, and SweissTek Viper MC was used as the plasma torch. The feedstock was a 1.2 mm-diameter low-carbon steel ER70S-6 wire with a chemical composition of Fe-0.1C-0.88Si-1.45Mn (wt.%).

**Figure 1:**
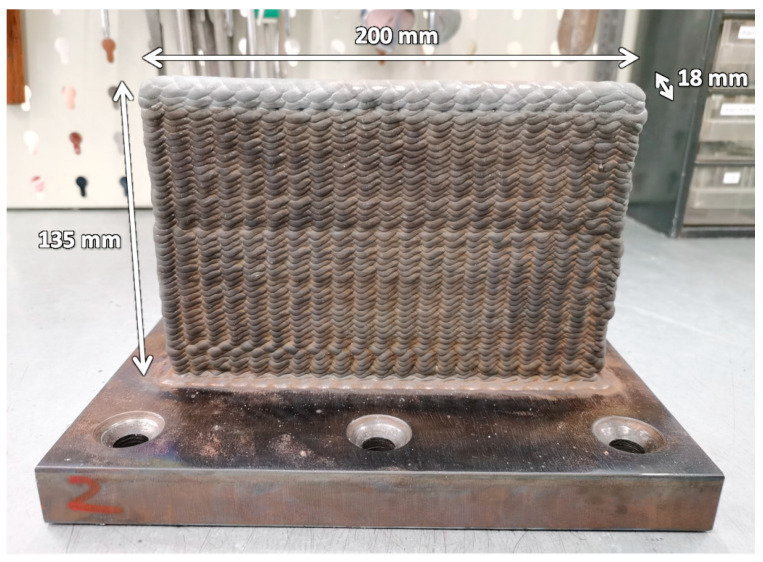
Dimensions of the WAAM-produced ER70S-6 steel.

For the printing parameters, an average current of 203 A, a voltage of 27 V, a wire feeding speed of 1.9 m/min, and a torch travelling speed of 3.8 mm/s were used. An oscillating printing path with a printing layer thickness of 2.5 mm was used. Argon gas was used for shielding. A single-wall part was produced with a length of 200 mm, a height of 135 mm, and a thickness of 18 mm, as shown in Figure 1.

### 2.2. Heat Treatment

After wire arc additive manufacturing, smaller size (15 mm × 10 mm × 4 mm) of samples were sectioned from the printed part for heat treatment processes. Six samples were heated at different temperatures (800 °C, 850 °C, 900 °C, and 950 °C) for different soaking durations (30 min, 60 min, and 90 min), as shown in Table 1. After the heating, all the samples were rapidly cooled to room temperature by water quenching. The different temperatures were used to study the effect of temperature on decarburization. For the same temperature of 900 °C, the heating duration was varied to study the effect of heating time on decarburization. The temperature of 900 °C was chosen for this study because it is a suitable austenitization temperature for heat treatment of the steel. 

### 2.3. Microstructure Characterization

Microstructure characterization was carried out at the cross-section planes of the heat-treated samples, especially in the region near the printed edges. Samples were mechanically ground and polished using the standard metallographic sample preparation technique. They were ground with SiC papers for up to 4000 grits and subsequently polished with 1 μm diamond suspension, OP-S, and then OP-U Struers colloidal silica suspensions. Samples were then etched with a 4% Nital solution (4% HNO_3_ nitric acid + 96% C_2_H_6_O ethanol) for about 10 s for microstructure observation.

Optical micrographs were obtained using the LEXT OLS4100 (Olympus, Tokyo, Japan) microscope. Secondary electron micrographs were obtained using a JOEL 5600 LV scanning electron microscope (SEM, JOEL, Tokyo, Japan) with an accelerating voltage of 10 kV. Electron backscattered diffraction (EBSD) maps were obtained using a field emission scanning electron microscope (FESEM) mounted with an Oxford Instruments detector and scanned using an accelerating voltage of 20 kV and a step size of 2 μm.

### 2.4. Microhardness and Nanohardness Test

Vickers microhardness tests were carried out using the Future-Tech FM-300e microhardness indenter machine (Future-Tech, Kawasaki, Japan) by applying the indentation load of 50 gf for a 10 s dwell time. Measurements with a depth interval of 50 μm and 100 μm were carried out. Five microhardness data were collected at each depth location, and the average microhardness as well as standard deviations were calculated.

For the as-printed and 800-30 heat-treated samples, in order to characterize the decarburization depths more accurately, nanohardness tests were carried out. Agilent G200 (Agilent Technologies, Santa Clara, CA, USA) nanoindenter machine equipped with a Berkovich indenter was utilized. A constant indentation depth limit of 300 nm with depth intervals of 20 μm (for the as-printed sample) and 50 μm (for the 800-30 sample) was set for the measurements. Three to five nanohardness data were collected at each depth location of the sample, and the average nanohardness and standard deviations were then calculated.

ASTM E1077-14 was used as the guideline [22]. Decarburization is deemed to have occurred when there is a significant drop in hardness measured near the edge surface of the material. The decarburization depth is defined as the distance from the edge surface to the location where the hardness levels.

### 2.5. Simulation Modelling

Numerical simulations on the carbon content change due to the diffusion of carbon during the heat treatment processes were carried out. The diffusion of carbon in austenite is determined by Fick’s second law of diffusion and can be expressed as [8]:

(1) $$J=-D\frac{\delta c}{\delta x}$$

where J is the net flux of carbon diffusing from a higher concentration region to a lower concentration region, D is the diffusion coefficient, which varies according to the concentration gradient of carbon and temperature, and $\frac{\delta c}{\delta x}$ is the concentration gradient of carbon. Thermo-Calc software with the diffusion module DICTRA was used to numerically solve the above carbon diffusion equation using the method developed by Andersson and Ågren [23]. The solution yields a carbon concentration profile at every step of the simulation. A simple one-dimensional model of a single-phase austenite region containing 0.1 wt.% C was used for the simulation. The heat treatment parameters are set to be the same as the parameters used in the experimental samples, as shown in Table 1. Carbon concentration profiles along the depth of the samples after the heat treatment processes were simulated.

## 3. Results and Discussion

### 3.1. Microstructure Analyses

**Figure 2:**
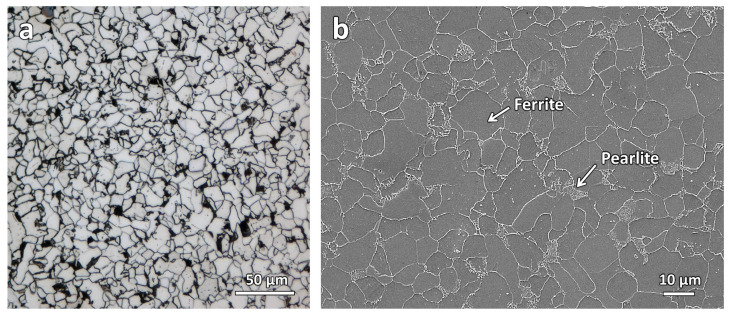
(**a**) Optical micrograph and (**b**) secondary electron micrograph of the WAAM-produced ER70S-6 steel.

Figure 2 shows the optical micrograph and the secondary electron micrograph of the as-printed sample. The micrographs show a typical low-carbon steel microstructure with a ferrite matrix and secondary pearlite phase [24].

**Figure 3:**
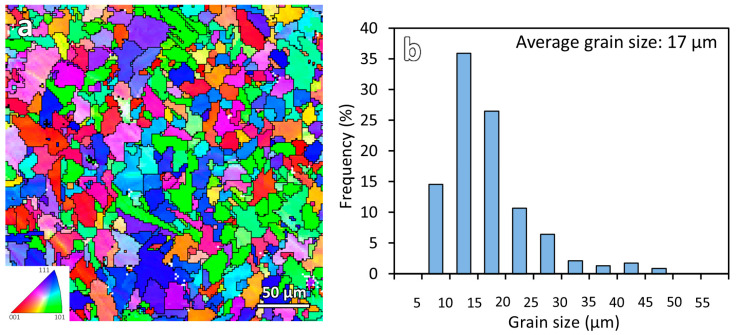
(**a**) Inverse pole figure (IPF-Y) map and (**b**) grain size distribution of WAAM-produced ER70S-6 steel.

Figure 3 shows the inverse pole figure (IPF-Y) map and the measured grain size distribution of the as-printed sample. From the IPF-Y map, the average grain size of the microstructure was measured using the EBSD post-processing HKL Channel 5 software (Oxford Instruments, Abingdon, UK). The average grain size of the as-printed ER70S-6 steel is 17 μm. All the IPF-Y maps in this study use the same color legend shown in Figure 3a.

**Figure 4:**
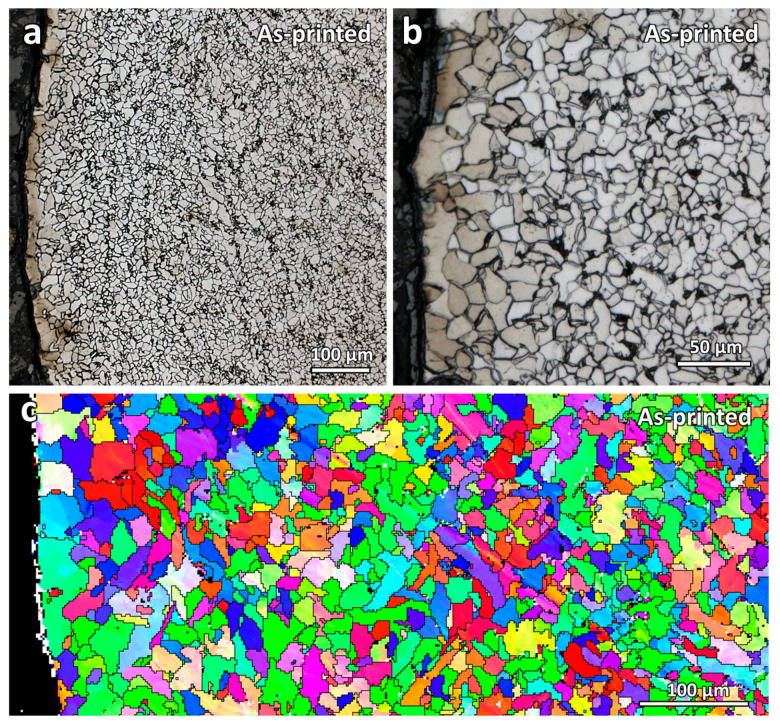
(**a**,**b**) Optical micrographs and (**c**) inverse pole figure (IPF-Y) map of the as-printed WAAM ER70S-6 steel.

Figure 4 shows the microstructure near the printed edge surface of the as-printed sample. As seen from the figure, a small decarburization layer can be observed in the as-printed sample. It is a layer that consists of larger ferrite grains in the absence of pearlite. Due to the carbon loss at this layer, grain growth is faster than that of the central region. Similar decarburization microstructure characteristics were also observed by Jing et al. [25] and Hayashi et al. [26].

**Figure 5:**
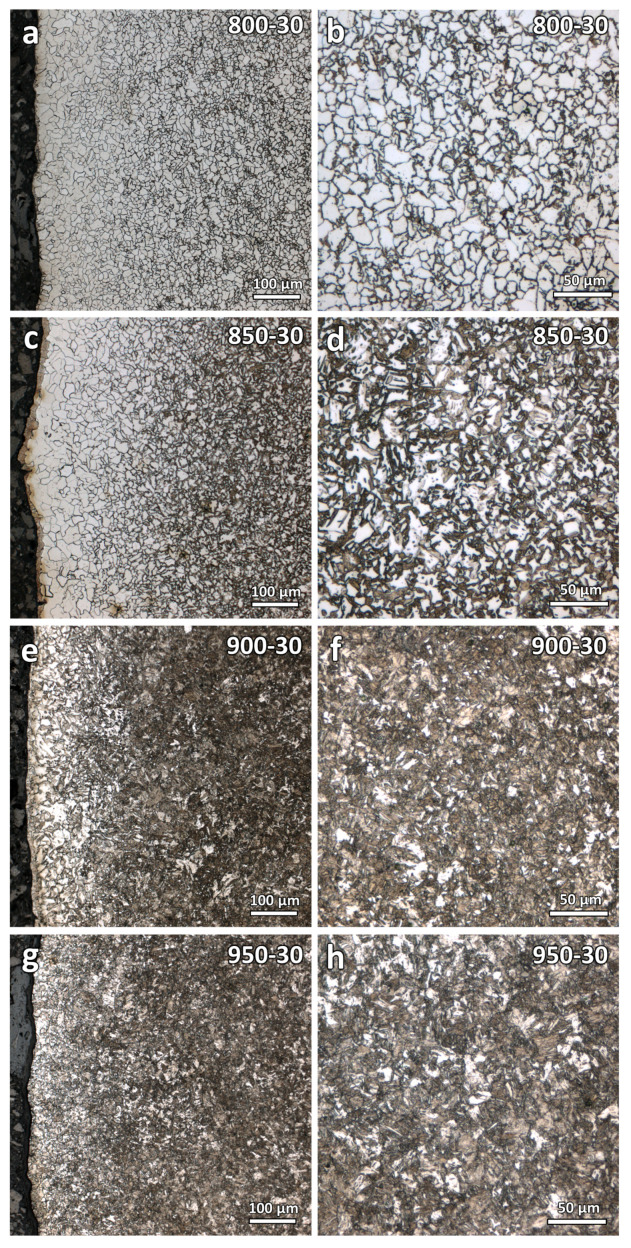
Optical micrographs at the near surface and central region of the varying temperature heat-treated samples: (**a**,**b**) 800-30, (**c**,**d**) 850-30, (**e**,**f**) 900-30, and (**g**,**h**) 950-30.

Figure 5 shows the optical micrographs at the near surface and central region of the varying temperature heat-treated samples (800-30, 850-30, 900-30, and 950-30). As seen from the figure, decarburization zones can be clearly observed near the printed edge surfaces. A noticeable difference in the microstructure can be observed. Carbon-loss layers with coarser ferrite grains were observed. However, as the samples were heat-treated at different temperatures, the resulting microstructures varied significantly. The sample that was heat-treated at a higher temperature was found to contain more martensite. For samples 900-30 and 950-30, the decarburization layer boundaries are difficult to determine. The decarburization depths of these four samples are difficult to quantitatively determine and compare using microstructure characterization.

**Figure 6:**
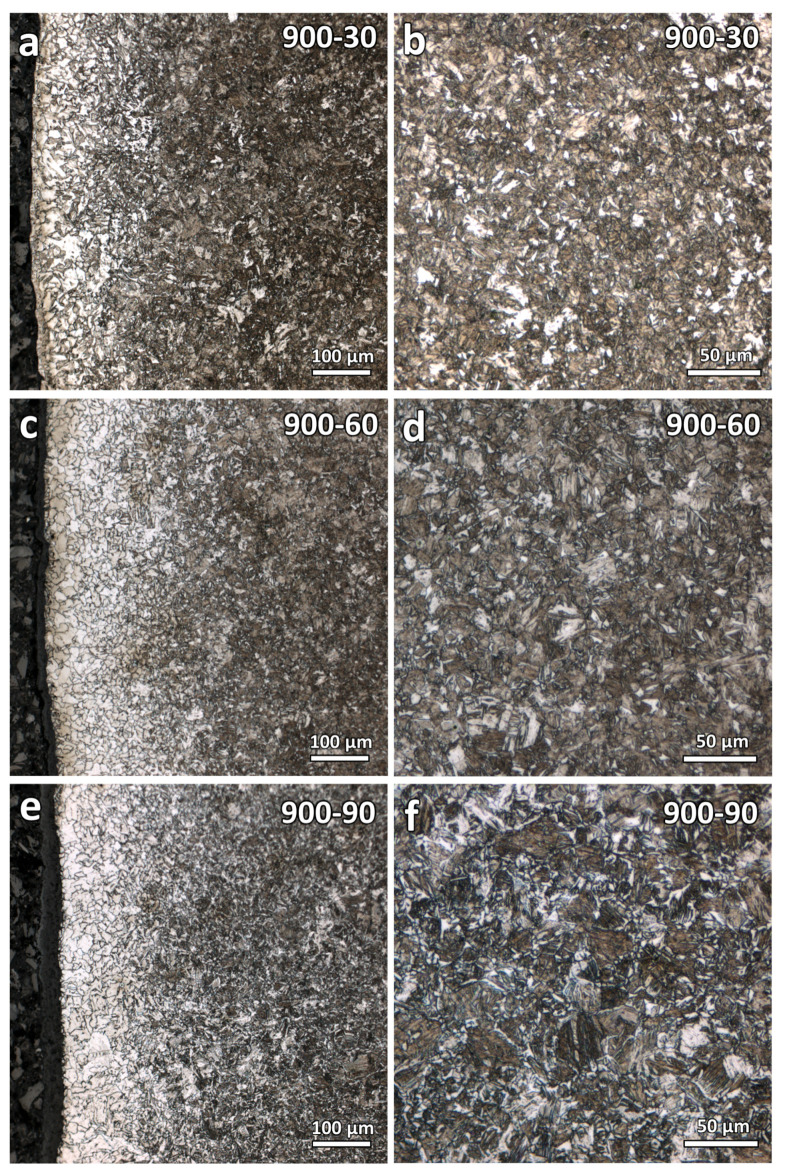
Optical micrographs at the near surface and central region of the varying duration heat-treated samples: (**a**,**b**) 900-30, (**c**,**d**) 900-60, and (**e**,**f**) 900-90.

Optical micrographs of the varying duration samples (900-30, 900-60, and 900-90) were obtained and shown in Figure 6. As seen from the figures, comparisons of the decarburization depths are more straightforward and can be conducted as the microstructures of these three samples are similar. They were heat-treated at the same temperature, only varying the soaking duration. It is observed that the decarburization depth increases with the increase in heat treatment soaking duration. However, the decarburization depths of these three samples are still difficult to quantitatively determine.

**Figure 7:**
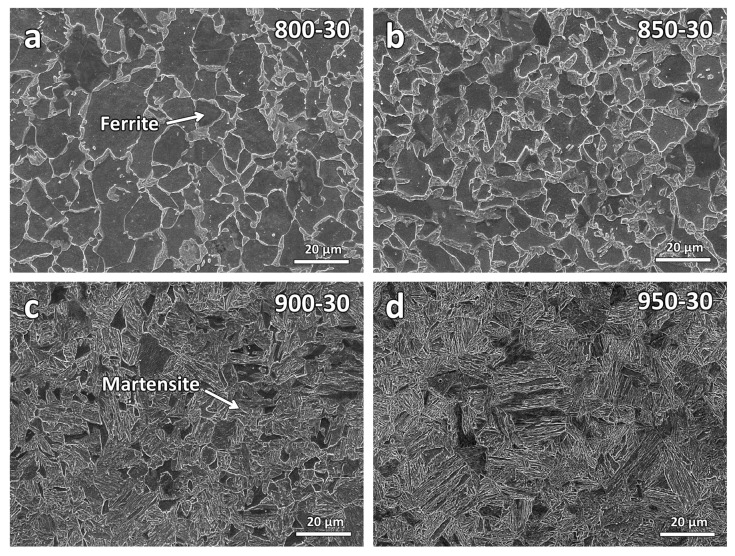
Secondary electron micrographs at the central region of the varying temperature heat-treated samples: (**a**) 800-30, (**b**) 850-30, (**c**) 900-30, and (**d**) 950-30.

To further evaluate the central region microstructures after the heat treatments, secondary electron micrographs of higher magnification were obtained. Figure 7 shows the SEM micrographs of the varying temperature samples. As seen from the figure, the volume fraction of the martensitic increases with the increase in temperature. This is expected as the heating temperatures are around the ferrite plus austenite region, and as the temperature increases, more austenite will form and transform to martensite during the quenching [27].

**Figure 8:**
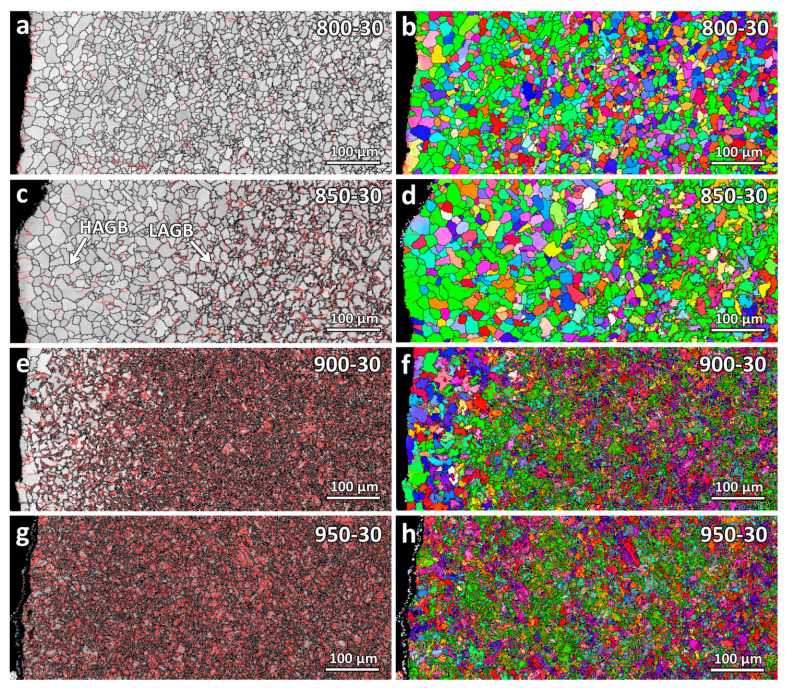
Grain boundary and inverse pole figure (IPF-Y) maps of the varying temperature heat-treated samples: (**a**,**b**) 800-30, (**c**,**d**) 850-30, (**e**,**f**) 900-30, and (**g**,**h**) 950-30.

EBSD scans were carried out on the varying temperature samples for further observation. The grain boundary maps and the inverse pole figure maps along the WAAM build-up direction (IPF-Y) are shown in Figure 8. All the samples were detected as having the body-centered cubic (BCC) crystal lattice structure, with no other lattice structures detected. For steels with a low carbon concentration of 0.1–0.6 wt.%, both ferrite and martensite are detected by EBSD as BCC lattice structure [28,29], although martensite is known to possess a body-centered tetragonal (BCT) lattice structure. 

In the grain boundary maps, black contours show the high-angle grain boundary HAGB (>10°), whereas red contours show the low-angle grain boundary LAGB (2–10°). As seen from the figure, a fraction of LAGB increases as the heating temperature increases. This is because of the martensitic microstructure formed in the samples. 

For the decarburization depth analysis, similar to the optical micrograph observation, the decarburization depths could not be clearly determined and compared using the EBSD maps. Therefore, in the next section, a further analysis of the decarburization depths will be carried out using the microhardness and nanohardness results instead.

### 3.2. Hardness Analyses

**Figure 9:**
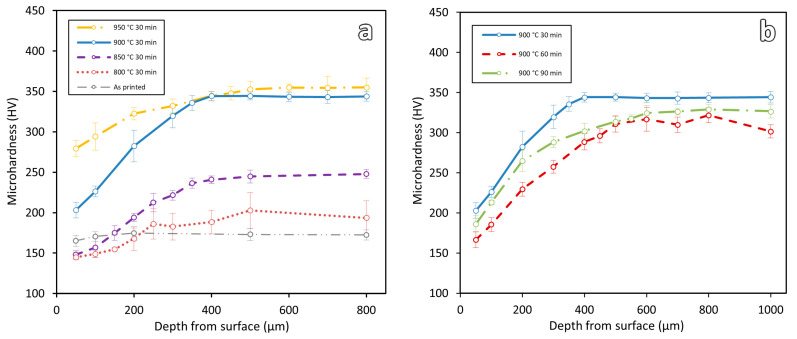
Microhardness results of the heat-treated WAAM samples for (**a**) varying temperatures and (**b**) varying soaking durations.

Figure 9 shows the consolidated microhardness results of all the heat-treated samples. Two comparison graphs were plotted for the varying temperature (Figure 9a) and duration (Figure 9b) of heat treatments. A distinct variation of hardness can be clearly observed along the material depth from the edge surface to the central region. Using these microhardness results, a quantitative value of decarburization depth can be found. 

As shown in Figure 9a, for the varying temperature samples, decarburization depths are observed to increase with the increase in temperature. Sample 800-30 has a decarburization depth of 250 μm, followed by 350 μm for sample 850-30, 400 μm for sample 900-30, and 500 μm for sample 950-30. Similarly, for the effect of soaking durations, decarburization depths are observed to increase with the increase in soaking duration. Sample 900-30 has a decarburization depth of 400 μm, followed by 500 μm for samples 900-60 and 600 μm for 900-90.

**Figure 10:**
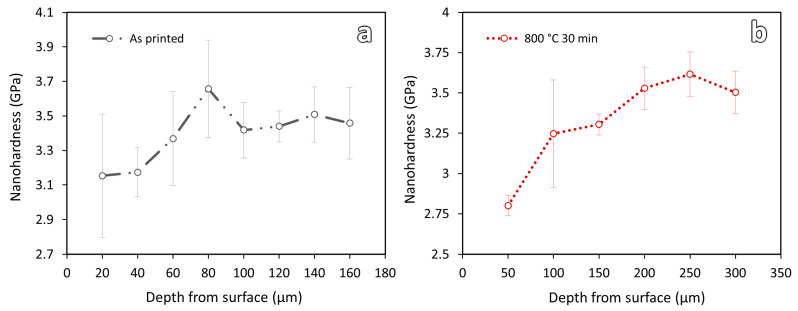
Nanohardness results of the (**a**) as-printed and (**b**) 800-30 sample.

For the as-printed and 800-30 samples, as their hardness values are relatively low and decarburization depths are smaller, nanohardness measurements were carried out to further verify the decarburization depths. Nanohardness test can give better spatial resolution. Figure 10 shows the nanohardness results of these two samples. As shown in the figure, a clear drop of hardness can be observed near the printed edge surfaces of the samples. The decarburization depths of the as-printed and 800-30 samples are observed to be about 60 μm and 200 μm, respectively.

### 3.3. Numerical Simulation Results

**Figure 11:**
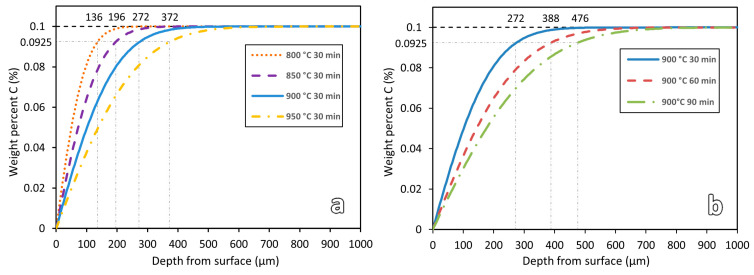
Simulation results of the heat-treated low-carbon steel samples for (**a**) varying temperatures and (**b**) varying soaking durations.

For comparisons, decarburization depths of a low-carbon steel of similar original C content exposed to similar heat treatment parameters were simulated. Carbon concentration profiles along the sample depth were obtained, as shown in Figure 11. The decarburization depth is defined as the distance from the edge surface to the location where 92.5% of the original carbon content is found. This is the threshold value at which decarburization depth was reported to be theoretically estimated with 97% accuracy [30,31].

As shown in Figure 11a, from the simulation results, for the varying temperature samples, decarburization depths were estimated to be 136 μm for sample 800-30, 196 μm for sample 850-30, 272 μm for sample 900-30, and 372 μm for sample 950-30. For the varying duration samples, sample 900-30 has a decarburization depth of 272 μm, followed by 388 μm for sample 900-60, and 476 μm for sample 900-60. Similar trends with the microhardness results are observed. The decarburization depth increases with the increase in heat treatment temperature or duration.

## 4. Discussion

### 4.1. Decarburization of WAAM-Produced ER70S-6 Steel

In this study, decarburization depths of the as-printed and heat-treated WAAM-produced ER70S-6 steel were evaluated using microstructure evaluations, hardness tests, and numerical simulation results. Results show that microstructure evaluations are not suitable for decarburization depth determination. At some microstructure states, it is difficult to observe and determine the decarburization depth (see the microstructures of samples 900-30 and 950-30 in Figure 5e,g).

However, hardness and simulation analyses are found to be useful for quantifying the decarburization depths of WAAM-printed parts. A distinct graduation change in the hardness profile can be observed in the decarburization zones. The decarburization depth of each sample can be quantitatively determined from the hardness profile. Using that, the decarburization depths of different samples can be determined and compared. For the simulation analyses, the decarburization depths are theoretically estimated based on the initial carbon content of the steel, the heat treatment temperatures, and the heat treatment soaking durations. The theoretical prediction of decarburization depth during the heat treatment has not been investigated for the decarburization prediction of WAAM-produced steel so far. Table 2 shows a summary of the decarburization depths obtained from the hardness and simulation results.

As shown in the table, the measured decarburization depths and those predicted by the numerical simulation show similar trends in terms of the effect of temperature or duration on the decarburization depth; however, the measured values are always higher than the simulation results, with the differences ranging from 64 μm to 154 μm. The discrepancy can be attributed to the initial decarburization condition of the steel before the heat treatment. The as-printed samples had a decarburization layer of about 60 μm (Figure 10). However, in the simulation work, the initial material is assumed to be free of decarburization. A more accurate prediction could be obtained from the numerical simulation if the initial decarburization depth was considered. 

### 4.2. Effect of Heat Treatment Temperature and Soaking Duration

**Figure 12:**
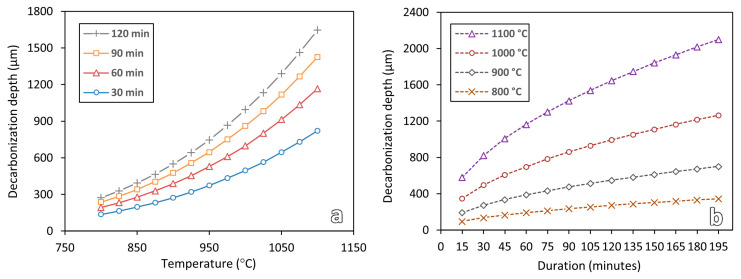
Simulation results for predicting the decarburization depths after heat treatment processes at different (**a**) temperatures and (**b**) soaking durations.

Both hardness and simulation results in Figure 9 and Figure 11 show that the decarburization depth increases with the increase in heat treatment temperature or duration. To further study the effect of temperature and soaking duration on decarburization, more simulations were conducted with wider ranges of temperatures (800 °C to 1100 °C) and durations (15 min to 195 min). The results are shown in Figure 12.

As shown in Figure 12, the influence of temperature keeps increasing as the temperature increases. However, for the soaking duration, the influence decreases as the soaking duration increases. Sample that is heat-treated at 1100 °C for merely 15 min, the decarburization depth is estimated to be about 581 μm. Whereas, for a sample that is heat-treated at 800 °C for a long duration of 195 min, the decarburization depth is estimated to be only about 344 μm. This shows that decarburization is more susceptible to temperature.

## 5. Conclusions

The decarburization of a low-carbon steel ER70S-6 produced by the WAAM process was investigated. Both as-printed and heat-treated samples were evaluated. Microstructure evaluations, hardness tests, as well as simulation works on the carbon diffusion process were carried out to evaluate the decarburization depths of the samples. It is shown that microstructure evaluation is not suitable for decarburization depth determination, and both hardness and simulation analyses are useful for quantifying decarburization of the WAAM-printed parts. Decarburization occurs not only during the heat treatment process but also during the printing process. The decarburization depth of the heat-treated WAAM-printed part was found to be a summation of decarburization during the printing and post-printing heat-treatment processes. Samples heat-treated for a short period of 30 min at 800 °C, 850 °C, 900 °C, and 950 °C were found to have decarburization depths of 200 μm, 350 μm, 400 μm, and 500 μm, respectively. For the fixed temperature of 900 °C, heating for durations of 30 min, 60 min, and 90 min resulted in decarburization depths of 400 μm, 500 μm, and 600 μm. The decarburization depth increases with the increase in heat treatment temperature or duration. 

## Figures and Tables

**Table 1:** Heat treatment parameters.

Temperature	Duration		
	30 min	60 min	90 min
800 °C	800-30		
850 °C	850-30		
900 °C	900-30	900-60	900-90
950 °C	950-30		

**Table 2:** Decarburization depths of as-printed and heat-treated (HT) samples.

Sample	Decarburization Depth Based on Hardness Results	Decarburization Depth Based on Simulation Results
As-printed	60 μm *	-
HT 800-30	200 μm *	136 μm
HT 850-30	350 μm	196 μm
HT 900-30	400 μm	272 μm
HT 950-30	500 μm	372 μm
HT 900-60	500 μm	388 μm
HT 900-90	600 μm	476 μm

* Based on nanohardness results.

## Data Availability

Not applicable.
